# Long coding RNA CCAT2 enhances the proliferation and epithelial-mesenchymal transition of cervical carcinoma cells via the microRNA-493-5p/CREB1 axis

**DOI:** 10.1080/21655979.2021.1969834

**Published:** 2021-09-09

**Authors:** Jing Wang, Yan Liu, Hongbing Cai, Hong Jiang, Wei Li, Yuying Shi

**Affiliations:** a Xiangyang Central Hospital, Affiliated Hospital of Hubei University of Arts and Science, P.R. China; b Department of Gynecological Oncology, Zhongnan Hospital of Wuhan University, Wuhan, P.R. China; c Hubei Key Laberatory of Tumor Biological Behavirs, P.R. China; d Hubei Cancer Clinical Study Center, P.R. China; e Department of Obstetrics and Gynecology, Xiangyang No.1 People’s Hospital, Hubei University of Medicine, Xiangyang, P.R. China

**Keywords:** Cervical carcinoma, CCAT2, miR-493-5p, EMT, CREB1

## Abstract

Cervical cancer (CC) is one of the most common malignancies among women. It has been demonstrated that long coding RNAs (lncRNAs) play a crucial role in CC. The purpose of this study was to investigate the role of the colon cancer associated transcript 2 (CCAT2) lncRNA in CC and elucidate its possible mechanisms of action. The expression of CCAT2, the miR-493-5p microRNA (miRNA), and mRNA was detected using qRT-PCR. Cell viability, proliferation, and migration and invasion were determined using the MTT, colony formation, and transwell assays, respectively. The interactions between miR-493-5p and CCAT2 or cAMP response element-binding protein 1 (CREB1) were verified using the luciferase and RNA pull-down assays. The effects of CCAT2 knockdown on *in vivo* tumor growth were determined using tumor xenografts and immunohistochemistry assays. The expression of CCAT2 was upregulated in CC cells and tissues. However, the knockdown of CCAT2 inhibited the proliferation and epithelial-mesenchymal transition (EMT) of CC cells *in vitro* and suppressed tumor growth *in vivo*. Mechanistically, CCAT2 functions as a competing endogenous RNA (ceRNA) to upregulate the expression of CREB1 by binding to miR-493-5p. The overexpression of CREB1 or downregulation of miR-493-5p antagonized the effect of CCAT2 knockdown on the proliferation and EMT of CC cells. The knockdown of CCAT2 suppressed the aggressiveness of CC via the miR-493-5p/CREB1 axis. Therefore, CCAT2 is likely to be a promising therapeutic target for CC.

## Introduction

Cervical cancer (CC) is a primary cancer of the cervix, that is usually caused by high-risk human papillomavirus (HPV) infections [1]. The symptoms of CC are not evident at an early stage, and most cases are diagnosed at an advanced stage of the disease [2]. At present, early CC can be detected by regular screening and prevented by vaccination; nevertheless, the prognosis of CC remains poor [3,4]. The treatment of CC includes traditional radiotherapy, chemotherapy, and immunotherapy [5]. However, radiotherapy and chemotherapy kill a considerable number of normal cells, and patients with CC are conducive to recurrence [6,7]. Moreover, distant metastasis alleviates clinical outcomes and exacerbates the progression of cancer. Therefore, identifying the specific therapeutic targets of CC is becoming increasingly important.

Long noncoding RNAs (lncRNAs) collectively participate in cancers by modulating multiple gene regulatory networks and gene transcription [8,9]. The colon cancer associated transcript 2 (CCAT2) lncRNA is encoded by a gene located on human chromosome 8 [8]. It has been reported that CCAT2 functions as an oncogene in multi-type cancers [9–13]. Lazniak et al. [14] reported that the CCAT2 rs6983267 single nucleotide polymorphism (SNP) is overexpressed in CC tissues. However, the precise mechanism underlying the role of CCAT2 in CC remains unknown.

Epithelial-mesenchymal transition (EMT) is characterized by the loss of epithelial features and acquisition of mesenchymal functions [15]. EMT contributes to the loss of cell-cell adhesion, apical-basal polarity, and cytoskeletal reorganization. These acquired mesenchymal characteristics further endow metastatic and stemness abilities to cancer cells [16]. Interestingly, the processes of EMT are highly dynamic and reversible. Numerous lncRNAs regulate the EMT of CC by modulating the expression of epithelial and mesenchymal markers. The CTS lncRNA enhances the transforming growth factor (TGF)-β1-induced-EMT of CC cells by downregulating E-cadherin and upregulating E-cadherin and vimentin [17]. However, the potential roles of CCAT2 in EMT remain to be elucidated.

In this study, the potential role of CCAT2 and underlying mechanism in CC was investigated. The effect of CCAT2 on the phenotype of CC cell lines was determined by functional studies.

## Materials and methods

### Clinical specimens

Thirty pairs of CC tissues and adjacent normal tissues were collected from the Zhongnan Hospital of Wuhan University. The study was approved by the ethics committee of Zhongnan Hospital of Wuhan University (201911210193). All the patients provided written informed consent. Criteria for inclusion: 1. CC tissues and adjacent normal tissues obtained by CC surgery and confirmed by pathological examination. 2. The patients did not receive radiotherapy, chemotherapy or biological immunotherapy before surgery. 3. Patients with severe liver and kidney diseases and diabetes were excluded.

### Cell culture and transfection

The cervical epithelial GH329 cell line and the HeLa, CaSki, Siha, and C4-1 CC cell lines were provided by the Shanghai Cell Bank of the Chinese Academy of Sciences. The cells were cultured in DMEM (11,965; Solarbio, China) supplemented with 10% FBS (10,270–106; Gibco, USA) and 1% penicillin/streptomycin at 37°C, in an atmosphere of 5% CO_2_ [18].

For this study, si-CCAT2, miR-493-5p mimic, miR-493-5p inhibitor, pcDNA3.1/CCAT2, pcDNA3.1/CREB1, pSilencer-sh-CCAT2, and the negative controls were obtained from GenePharma (Shanghai, China). The cells were transfected using Lipofectamine 3000 (Thermo Fisher Scientific, USA).

### qRT-PCR

The total RNA was isolated from the tissues and cells, and the cDNA was synthesized using a PrimeScript RT reagent kit (TaKaRa, Japan) [19]. PCR was conducted using SYBR® Premix Ex Taq™ (TaKaRa, Dalian, China). The results were obtained using the 2^−ΔΔCt^ method. U6 and GAPDH served as the internal controls.

### Western blotting assay

Total protein was collected using the RIPA buffer (Beyotime, China). Protein concentration was measured using a bicinchoninic acid kit. The proteins (15 µg) were then isolated using 10% SDS-PAGE and transferred onto PVDF membranes (Millipore, USA). After sealing with 5% nonfat milk, the membranes were incubated with primary antibodies and with horseradish peroxidase-conjugated secondary antibodies. Protein bands were detected using an ECL reagent (EMD Millipore, USA) [20]. Primary antibodies were all bought from Abcam (USA) and were listed as followed: anti-cyclin E (1/1000, ab33911), anti-ki67 (1/5000, ab92742), anti-N-cadherin (1/5000, ab76011), anti-E-cadherin (1/10,000, ab40772), and anti-vimentin (1/1000, ab92547), and GADPH (1/1000, ab8245).

### MTT assay

The viability of CC cells was measured using the MTT assay (ST316; Beyotime, China). The cells were collected and cultured in an incubator, following which they were treated with 20 μL MTT and cultured for 4 h. Then, 150 μL of DMSO (2206–27-1; Aladdin, China) was added to each well for dissolving the formazan. The absorbance of each well was measured at 450 nm using a microplate reader (Bio-Rad, USA) [21].

### Colony formation assay

Following transfection, the cells were suspended and inoculated at 37°C in an atmosphere of 5% CO_2_. The cells were then cultured with 2 ml culture medium for 2–3 weeks. The culture was terminated following the formation of a visible clone in the culture dish. The cells were subsequently washed with PBS (P1020; Solarbio, China) and stained with Giemsa stain. The clones were counted under a microscope (Olympus, Tokyo, Japan) [22].

### Transwell assay

The cells were collected following transfection, and 10 μL of the cell suspension was seeded into a 24-well plate (BD company, USA) at a density of 1.25 × 10^5^/mL. The inoculated cells were placed in an incubator and cultured for 24 h at 37°C in an atmosphere of 5% CO_2_ [23]. The cells were stained with the Giemsa kit (Abcam, Shanghai, China). Images of the cells were subsequently captured and the cells were counte.

### Dual luciferase reporter assay

The targets of CCAT2 and miR-493-5p were predicted using the online miRNA target prediction databases, TargetScan 7.2 (http://www.targetscan.org/mmu_72/) and miRDB (http://www.mirdb.org/), respectively [24]. The putative binding sites between miR-493 and the 3'-UTR of CCAT2 or CREB1 were inserted into a pmirGLO vector (Promega, USA). The cells were subsequently co-transfected with the miR-493 mimics or negative control (nc) mimics, and the 3'-UTR of wild-type (WT) or mutant (MUT) CCAT2 (or 3'-UTR of WT or MUT CREB1), using Lipofectamine 3000 [25]. The results were determined using a luciferase assay kit (Promega, USA).

### RNA pull-down assay

The miR-493 pull-down assay was performed as previously described [26]. Briefly, biotinylated negative control and miR-493 were acquired from GenePharm (Shanghai, China). Hela and Siha cells were cultured with either control probe or miR-493 probe and incubated with streptavidin-coupled beads. Subsequently, the precipitated RNA was eluted. The results were analyzed by qRT-PCR.

### Xenograft assay

Six-week-old male BALB/c nude mice were purchased from the Guangdong Medical Laboratory Animal Center (China), and divided into pSilencer group (n = 3) as well as pSilencer/sh-CCAT2 group (n = 3). HeLa cells (5 × 10^6^) stably expressing the pSilencer vector or pSilencer/sh-CCAT2 were suspended and injected into the dorsal aspect of the mice [27]. After 7 days, the weight and volume of the tumors were determined by measuring the width and length of the tumors every two days. The tumor volume was calculated using the following formula: tumor volume = ½ × (width)^2^ × length. The mice were subsequently euthanized by the intraperitoneal injection of pentobarbital (160 mg/kg). The entire duration of the animal studies, from injection to euthanasia, lasted for 45 days. This study was approved by the ethics committee of Zhongnan Hospital of Wuhan University (201908050082).

### Immunohistochemistry (IHC) studies

The paraffin-embedded tumor tissues were sliced into 4 μm-thick sections. The tissues sections were then deparaffinized in xylene and subsequently rehydrated. Hydrogen peroxide (0.5%) was used for blocking the endogenous peroxidase activity. The tissue sections were incubated overnight with a specific primary antibody against ki67 (1 µg/mL; ab15580; Abcam, USA) at 4°C [19]. The nuclei were counterstained with DAPI (D9564; Sigma, USA), and the stained slides were photographed under a microscope (Nikon, Japan).

### Statistical analyses

All the independent experiments were conducted in thrice. The data were analyzed using SPSS version 25.0. The data are presented as the mean ± standard deviation (SD). The differences were analyzed using Student’s t-test or one-way analysis of variance (ANOVA) followed by Tukey’s post-hoc test. Statistical significance was considered at *p* < 0.05.

## Results

### CCAT2 was upregulated in CC cells

**Figure 1.**
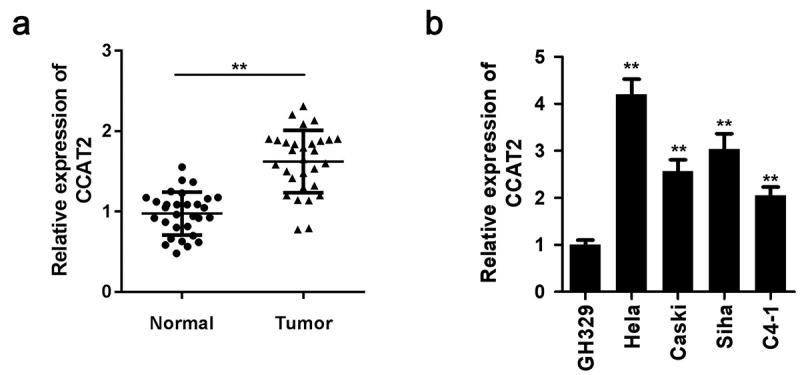
The expression of CCAT2 increases in cervical cancer (CC). (a) The expression of CCAT2 in the clinical samples was detected by PCR. (b) The expression of CCAT2 in CC cells was detected by qRT-PCR. **p < 0.01, compared with Normal group and GH329 group

The expression levels of CCAT2 in the clinical samples were evaluated using qPCR. The expression of CCAT2 was markedly upregulated in the human CC tissues (Figure 1a). We then determined the expression of CCAT2 in the HeLa, CaSki, Siha, and C4-1 CC cell lines. As depicted in Figure 1b, the expression of CCAT2 dramatically increased in CC cells, and the expression was higher in HeLa cells and SiHa cells, compared to that in the other CC cell lines (Figure 1b).

### CCAT2 knockdown inhibited the proliferation and EMT of CC cells

**Figure 2.**
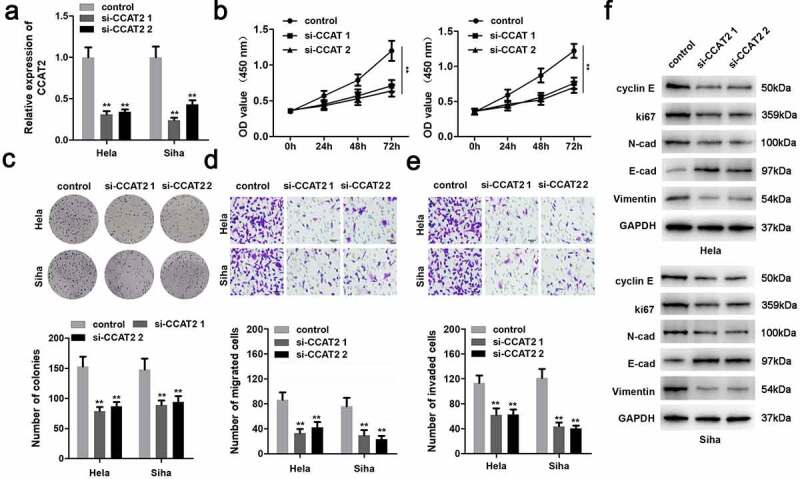
Knockdown of CCAT2 inhibits the proliferation and epithelial-mesenchymal transition (EMT) of CC cells. (a) The expression of CCAT2 detected by PCR. (b) Cellular viability determined using the MTT assay. (c) Cellular proliferation measured using the colony formation assay, for detecting the proliferation of HeLa and Siha cells. (d and e) Cellular migration and invasiveness determined using the transwell assay. (f) The expression of cellular proteins determined by western blotting. **p < 0.01, compared with control group

CCAT2 was notably downregulated in the cells transfected with si-CCAT2 1# or si-CCAT2 2# (Figure 2a). Moreover, the knockdown of CCAT2 significantly suppressed the proliferation of CC cells (Figure 2b and c). This observation was consistent with the results of the transwell assay. As depicted in Figure 2d and e, the knockdown of CCAT2 markedly inhibited the migration and invasiveness of CC cells. Moreover, the downregulation of CCAT2 increased the expression of E-cadherin protein and decreased the expression of cyclin E, Ki-67, N-cadherin, and vimentin (Figure 2f).

### CCAT2 inhibited the growth of CC cells in vivo

**Figure 3.**
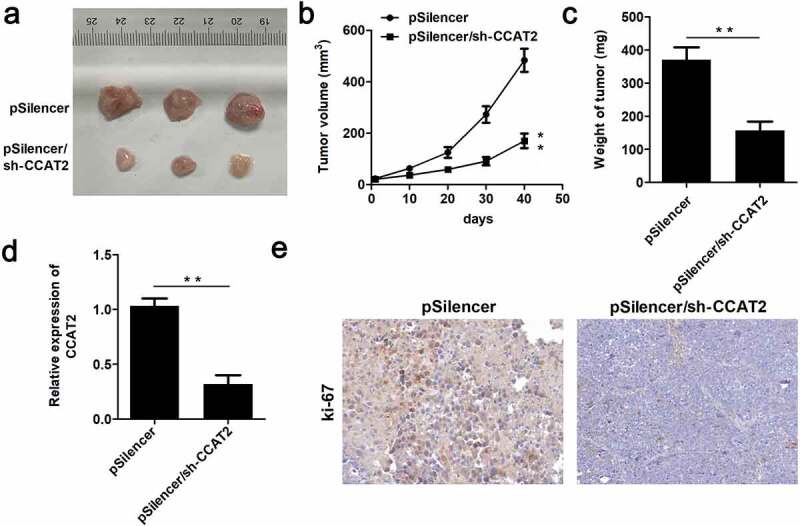
CCAT2 inhibits the growth of CC cells in nude mice. HeLa cells were transfected with psilencer, and psilencer/CCAT2 was separately injected into the dorsal aspect of 6-week-old nude mice. (a) The tumors in the two groups are depicted. (b) Plot showing the survival curve of the tumors. (c) The weights of the tumors were measured. (d) The expression of CCAT2 in the tumors detected by qPCR. (e) The expression of ki-67 in the tumors assessed by immunohistochemistry (IHC) studies. **p < 0.01, compared with pSilencer group

In order to further verify the inhibitory potential of CCAT2 in CC, we performed an *in vivo* assay. As depicted in Figure 3a–c, CCAT2 significantly suppressed tumor growth and decreased the volume and weight of the tumors. Moreover, the expression of CCAT2 was significantly downregulated in the CCAT2-knockdown group (Figure 3d). The results of IHC studies revealed that the expression of ki-67 decreased in the CCAT2-knockdown group (Figure 3e).

### CCAT2 sponges miR-493-5p in CC

**Figure 4.**
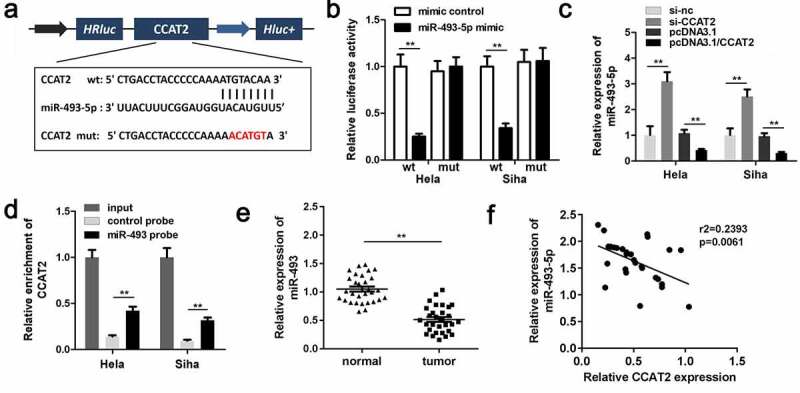
CCAT2 sponges miR-493-5p in CC cells. (a) The predicted binding sequence between CCAT2 and miR-493-5p. (b) The luciferase activity detected using the luciferase assay. (c) The expression of miR-493-5p detected by PCR. (d) The interactions between CCAT2 and miR-493-5p determined using RNA pull-down assays. (e) The expression of miR-493-5p in the clinical samples determined by PCR. (f) The correlation between the expression of CCAT2 and miR-493-5p detected by Pearson analysis. **p < 0.01, compared with mimic control group, si-nc group, pcDNA3.1 group, control probe, and normal group

The predicted binding sequences between miR-493-5p and CCAT2 are depicted in Figure 4a. Moreover, co-transfection with the miR-493-5p mimic and 3'-UTR of WT CCAT2 remarkably reduced the luciferase activity (Figure 4b). The expression of miR-493 was downregulated by CCAT2 in CC cells (Figure 4c). The RNA pull-down assay further confirmed the interaction between miR-493-5p and CCAT2 (Figure 4d). The results demonstrated that miR-493-5p was notably downregulated in the CC tissues (Figure 4e). The results of Pearson analysis revealed that the expression of miR-493-5p was negatively correlated with the expression of CCAT2 (Figure 4f). The results demonstrated that CCAT2 directly targeted miR-493-5p in HeLa and SiHa cells.

### Downregulation of miR-493-5p promoted the aggressiveness of CC cells

**Figure 5.**
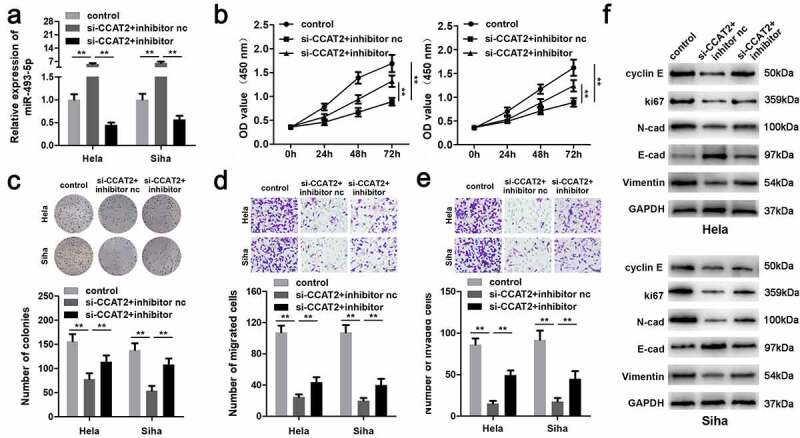
Downregulation of miR-493-5p increases the aggressiveness of CC. (a) The expression of miR-493-5p as detected by PCR. (b) Cellular viability determined using the MTT assay. (c) Cellular proliferation determined using the colony formation assay. (d and e) Cellular migration and invasiveness detected using the transwell assay. (f) The expression of cellular proteins determined by western blotting. **p < 0.01, compared with control group and si-CCAT2+ inhibitor nc group

The results demonstrated that miR-493-5p was significantly downregulated by the miR-493-5p inhibitor, suggesting successful transfection (Figure 5a). The downregulation of miR-493-5p alleviated the effects of CCAT2 knockdown and promoted the proliferation of CC cells (Figure 5b and c). These results were consistent with the results of the transwell assay. As depicted in Figure 5d and e, the inhibition of miR-493-5p significantly suppressed the migration and invasiveness of CC cells, compared to those of the si-CCAT2 + nc inhibitor group. Moreover, the downregulation of miR-493-5p reversed the effects of CCAT2 knockdown on the expression of cyclin E, Ki-67, N-cadherin, vimentin, and E-cadherin (Figure 5f).

### miR-493-5p directly targeted CREB1 in CC

**Figure 6.**
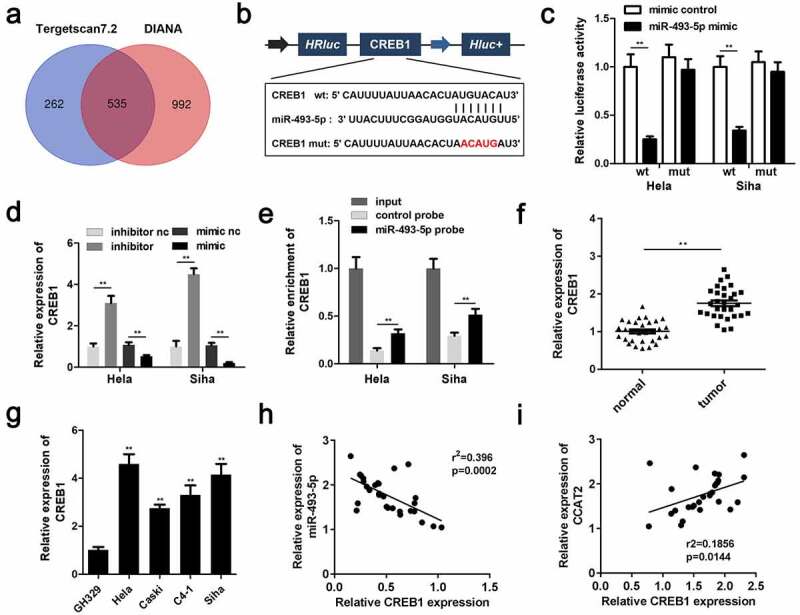
miR-493-5p targets CREB1 in CC cells. (a) The predicted target of miR-493-5p. (b) The predicted binding sites between CREB1 and miR-493-5p. (c) The luciferase activity detected using the luciferase assay. (d) The mRNA expression of CREB1, as detected by PCR. (e) The interactions between CREB1 and miR-493-5p verified using the RNA pull-down assay. (f) The mRNA expression of CREB1 in the clinical samples, as detected by qPCR. (g) The expression of CREB1 in the CC cells, as detected by PCR. (h, i) The correlation between the expression of CREB1 and that of CCAT2 or miR-493-5p, as determined by Pearson analysis. **p < 0.01, compared with mimic control group, inhibitor nc group, mimic nc group, control probe group, normal group, and GH329 group

The targets of miR-493-5p were predicted using the online miRNA target prediction databases, TargetScan (release 7.2) and miRDB, and a total of 535 genes were predicted by both the databases (Figure 6a). Figure 6b illustrated the predicted target regions between miR-493-5p and CREB1. The luciferase activity of the CC cells transfected with the miR-493-5p mimic and the 3'-UTR of WT CREB1 was significantly decreased (Figure 6c). CREB1 was significantly upregulated by the miR-493-5p inhibitor and downregulated by the miR-493-5p mimic (Figure 6d). Additionally, the RNA pull-down assay further confirmed the interactions between miR-493-5p and CREB1 (Figure 6e). The expression of CREB1 was markedly upregulated in the CC tissues and cells (figure 6f,g). The expression of miR-493-5p negatively correlated with the expression of CREB1 (Figure 6h), whereas expression of CCAT2 positively correlated with the expression of CREB1 (Figure 6i).

### CREB1 promoted the proliferation and EMT of CC cells

**Figure 7.**
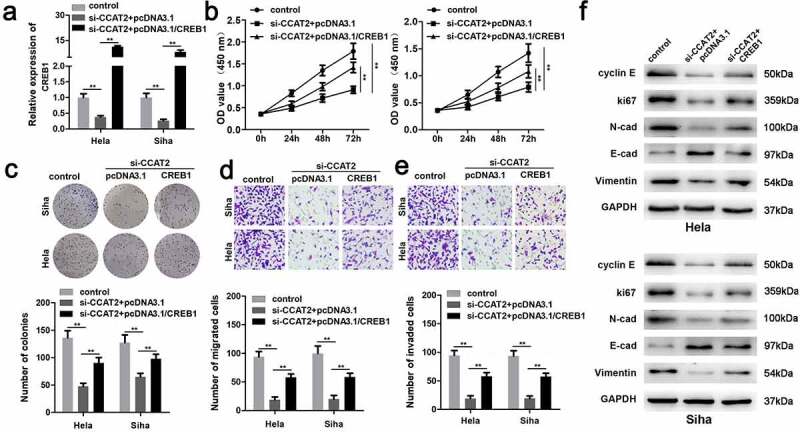
The overexpression of CREB1 reverses the effects of CCAT2 knockdown. (a) The mRNA expression of CREB1, as detected by PCR. (b) Cellular viability determined using the MTT assay. (c) Cellular proliferation determined using the colony formation assay. (d and e) Cellular migration and invasiveness detected using the transwell assay. (f) The expression of cellular proteins determined by western blotting. **p < 0.01, compared with control group and si-CCAT2+ pcDNA3.1 group

The mRNA expression of CREB1 was significantly upregulated in the pcDNA3.1/CREB1 group (Figure 7a). The overexpression of CREB1 markedly induced the proliferation of HeLa and SiHa cells (Figure 7b and c). The upregulation of CREB1 significantly enhanced the migration and invasiveness of CC cells (Figure 7d and e). Additionally, the overexpression of CREB1 decreased the expression of E-cadherin and increased the expression of cyclin E, Ki-67, N-adherin, and vimentin (Figure 7f).

## Discussion

In this study, the CCAT2 lncRNA was found to be overexpressed in CC tissues and cells. Interestingly, the knockdown of CCAT2 inhibited the proliferation and EMT of CC cells both *in vivo* and *in vitro*. Moreover, CCAT2 functioned as a ceRNA for upregulating the expression of CREB1 by sponging miR-493-5p, and the CCAT2/miR-493-5p/CREB1 axis regulated the malignant behavior of CC cells.

The CCAT2 lncRNA is encoded by a gene located at human chromosomal region 8q24, which is associated with the occurrence of colon cancer. It has been reported that the expression of CCAT2 significantly increases tumor-node-metastasis (TNM) and is associated with TNM, and may be a potential diagnostic biomarker for colorectal cancer. CCAT2 partakes in the development of colon cancer by regulating MYC and Wnt [28]. Increasing evidence has verified the potential of CCAT2 in human cancers. A high level of CCAT2 in gliomas is predictive of poor clinical results. CCAT2 promotes the growth and metastasis of breast cancer. The silencing of CCAT2 attenuates the proliferation, cell cycle, and migration of glioma cells. Yu et al. verified that CCAT2 promotes the progression of esophageal carcinoma [29]. Huang et al. revealed that CCAT2 promotes tumorigenesis in renal cell carcinoma, both *in vivo* and *in vitro* [30]. Additionally, the overexpression of CCAT2 correlates with poor prognosis in squamous cell carcinoma of the cervix [31]. The knockdown of CCAT2 suppresses the malignant behaviors of CC cells. In our study, the CCAT2 lncRNA was found to be overexpressed in CC, and the knockdown of CCAT2 suppressed the EMT of CC cells. EMT is accompanied by the degradation of epithelial features and acquisition of mesenchymal characteristics [32]. Moreover, EMT promotes the migration and invasivenss of cancer cells, which promotes the metastasis of CC [17]. The knockdown of CCAT2 upregulated the expression of E-cadherin and downregulated the expression of cyclin E, N-cadherin, and vimentin, which in turn suppressed the distant metastasis of CC cells. Altogether the results demonstrated that the knockdown of CCAT2 suppressed the aggressive phenotype of CC cells.

However, the role of CCAT2 in cancer is complicated. CCAT2 regulates the metabolism of cancer cells by binding to the cleavage factor I (CFIm) complex [33]. The E2F1-induced activation of CCAT2 enhances the interaction of CCAT2 with PTTG1 to promote the progression of pituitary adenomas [34]. Moreover, it is known that lncRNAs act as miRNA sponges to regulate gene expression. CCAT2 binds to miR-145 and miR-424 in colon cancer and gliomas, respectively [29,35]. The results of this study demonstrated that CCAT2 sponged miR-493-5p in the CC cells and tissues. It has been reported that miR-493-5p is downregulated in CC, which is consistent with the results of the present study [36]. The downregulation of miR-493-5p promoted the proliferation and EMT of CC cells; however, the underlying molecular mechanisms remain unclear.

It is known that miRNAs partake in biological processes by degrading the expression of their target(s) [37]. The results of this study demonstrated that miR-493-5p directly targeted CREB1. CREB1 plays an oncogenic role in various cancers, including bladder, lung, breast, and colorectal cancers. In CC, CREB1 promotes the proliferation of CC cells by inhibiting the expression of VDAC1 [38]. The present study demonstrated that CREB1 was upregulated in CC, and the overexpression of CREB1 alleviated the effects of CCAT2 and promoted the proliferation and EMT of CC cells. These results suggested that CCAT2 partakes in the development of CC via the miR-493-5p/CREB1 axis.

However, there are some limitations in this study. The number of clinical samples included is small and only Han patients were included. In future studies, the sample size should be expanded and multi-ethnic samples should be added.

## Conclusion

The results of this study demonstrated that the expression of CCAT2 was upregulated in CC. CCAT2 functions as an onco-lncRNA in CC by regulating the miR-493-5p/CREB1 axis. The findings of this study are likely to provide novel therapeutic targets and strategies for CC.

## Data Availability

The data that supports the findings of this study are available in the supplementary material of this article.

## Research highlights

1. LncRNA CCAT2 was overexpressed in the CC tissues and CC cell lines.
2. Knockdown of CCAT2 inhibited the proliferation and epithelial-mesenchymal transition (EMT) of CC cells in vitro and suppressed tumor growth in vivo.
3. The knockdown of CCAT2 suppressed the aggressiveness of CC cells via the miR-493-5p/CREB1 axis.
